# Transcutaneous Spinal Cord Stimulation Enhances Quadriceps Motor Evoked Potential in Healthy Participants: A Double-Blind Randomized Controlled Study

**DOI:** 10.3390/jcm9103275

**Published:** 2020-10-13

**Authors:** Álvaro Megía-García, Diego Serrano-Muñoz, Julian Taylor, Juan Avendaño-Coy, Natalia Comino-Suárez, Julio Gómez-Soriano

**Affiliations:** 1 Biomechanical and Technical Aids Unit, National Hospital for Paraplegia, SESCAM, 45071 Toledo, Spain; amegiag@externas.sescam.jccm.es; 2 Toledo Physiotherapy Research Group (GIFTO), Faculty of Physiotherapy and Nursing, Castilla La Mancha University, 45071 Toledo, Spain; juan.avendano@uclm.es (J.A.-C.); natalia.comino@cajal.csic.es (N.C.-S.); julio.soriano@uclm.es (J.G.-S.); 3 Sensorimotor Function Group, National Hospital for Paraplegia, SESCAM, 45071 Toledo, Spain; juliantaylorgreen2@gmail.com; 4 Harris Manchester College, University of Oxford, Oxford OX1 3TD, UK; 5 Neural Rehabilitation Group, Cajal Institute, Spanish National Research Council (CSIC), 28002 Madrid, Spain

**Keywords:** transcutaneous spinal cord stimulation, evoked potentials motor, neuromodulation, motor activity

## Abstract

Transcutaneous electrical spinal cord stimulation (tSCS) is a non-invasive technique for neuromodulation and has therapeutic potential for motor rehabilitation following spinal cord injury. The main aim of the present study is to quantify the effect of a single session of tSCS on lower limb motor evoked potentials (MEPs) in healthy participants. A double-blind, sham-controlled, randomized, crossover, clinical trial was carried out in 15 participants. Two 10-min sessions of tSCS (active-tSCS and sham-tSCS) were applied at the T11-T12 vertebral level. Quadriceps (Q) and tibialis anterior (TA) muscle MEPs were recorded at baseline, during and after tSCS. Q and TA isometric maximal voluntary contraction was also recorded. A significant increase of the Q-MEP amplitude was observed during active-tSCS (1.96 ± 0.3 mV) when compared from baseline (1.40 ± 0.2 mV; *p* = 0.01) and when compared to sham-tSCS at the same time-point (1.13 ± 0.3 mV; *p* = 0.03). No significant modulation was identified for TA-MEP amplitude or for Q and TA isometric maximal voluntary isometric strength. In conclusion, tSCS applied over the T11-T12 vertebral level increased Q-MEP but not TA-MEP compared to sham stimulation. The specific neuromodulatory effect of tSCS on Q-MEP may reflect optimal excitation of this motor response at the interneuronal or motoneuronal level.

## 1. Introduction

Transcutaneous electrical spinal cord stimulation (tSCS) is a non-invasive technique designed to generate neuromodulation of the central nervous system at the spinal cord level. During the last ten years, studies have reported changes in excitability of neural networks organized at the spinal cord level by transcutaneous electrical stimulation applied via surface electrodes placed over the middle of back (T10-T11 spinal level) [1,2]. At the spinal level, tSCS modulates the excitability of sensorimotor circuits which are also affected by epidural stimulation [3]. Both techniques can evoke posterior root muscle (PRM) reflex responses measured at the target muscle [3]. The low PRM reflex threshold response to stimulation, suggests that tSCS activates large-to-medium diameter afferent fibers (Ia, Ib, II) within the sensory dorsal roots [1,3]. The similarity between PRM and Hoffmann reflex responses also suggests that tSCS activates proprioceptive Ia afferent fibers [4,5].

Several studies have also revealed that tSCS mediates several therapeutic effects in subjects with neurological impairments [6]. Thus, tonic tSCS, applied as a biphasic rectangular current at a frequency of 30–50 Hz, has shown a therapeutic potential to enhance voluntary motor activity [7,8], trunk stability [9], standing [10], gait function [7,11,12] and as a method to reduce spasticity [13,14]. However, the clinical effectiveness of this technique is still undetermined, and the stimulation parameters need to be optimized [6].

The results of a recently published review by our group, Megía-García et al. [6], suggest that the optimal site for spinal stimulation to enhance voluntary motor control of the lower extremities is over the T11-T12 vertebrae, which corresponds to the L1-L2 spinal cord segments. The idea of the existence of the central pattern generation (CPG) at this level [15], together with a multisegmental muscle activity in response to stimulation applied at this level [16], suggests that activation of local or propriospinal motor control mechanisms potentiate descending corticospinal acting on spinal motoneurons, thereby mediating the neuromodulatory effect of tSCS leading to improve motor function following spinal cord injury [15]. However, studies performed on healthy subjects have shown that the optimal stimulation site differs according to which target muscle group is activated [17,18,19]. Hence, greater activation of the anterior rectus femoris and vastus lateralis has been observed with T10-T11 (L1-L2 spinal cord segments) stimulation when compared to the hamstring, triceps surae, and tibialis anterior muscles which are activated optimally with T12-L1 stimulation (L5-S1 spinal cord segments) [19,20]. In line with these observations, the phenomenon of spatial summation has also been reported, with greater motor responses recorded from the target muscle with simultaneous electrical stimulation of contiguous spinal levels [21].

Although tSCS has been shown to be a safe technique with therapeutic potential for motor function rehabilitation following neurological diseases [6], it is necessary to understand how the tSCS technique can be optimized in the clinical practice. The validation of neurophysiological measurements which reflect changes in both cortical and spinal excitability, such as motor evoked potentials (MEPs) evoked by transcranial magnetic stimulation (TMS), and closer attention to appropriate sham-protocols are important first steps for developing future tSCS studies related to better neuromodulation with optimized stimulation parameters and electrode locations.

Until now, tSCS-mediated neuromodulation of the motor response has been measured indirectly through electromyogram recordings of ongoing voluntary muscle activity in combination with dynamometry, manual muscle testing, kinematics and functional assessments [7,8,22,23]. However, these measurement tools have a low sensitivity to single session tSCS-induced neuromodulation and cannot detect small changes in neuromuscular activity. Neurophysiological methods aimed at measuring changes in spinal excitability, such as the PRM reflex and the Hoffman reflex, have been used to assess neuromodulation after tSCS [24,25,26,27]. However, the extent to which tSCS can lead to a change in cortical and spinal excitability has only been studied in the upper limb [27]. Therefore, the measurement of MEPs evoked by TMS has been used in this study as the standard and sensitive neurophysiological method to assess changes in excitability of the corticospinal pathway in response to tSCS.

The main aim of the present study is to quantify the effect of a single session of active T11-T12 tSCS on lower limb MEP amplitude measured from the quadriceps and tibialis anterior muscles of healthy participants. We hypothesized that a single session of tSCS would increase corticospinal excitability with a preferential effect on those motoneurones closer to the level of spinal stimulation. As a secondary aim the efficacy of blinding both the sham and active tSCS was carefully analyzed using specialized metrics appropriate to randomized control trials.

## 2. Experimental Section

The present study was approved by the local Toledo Ethical Committee (Ref. No. 158; 2/11/2017) and the clinical trial was registered in the ClinicalTrials.gov Protocol Registration System (NCT04241406). Participants were informed about the protocol and signed the informed consent approved by the local ethics committee.

### 2.1. Design

A double-blinded, randomized, controlled, crossover clinical trial was designed. All participants (*n* = 15) received two randomized sessions of tSCS stimulation (active-tSCS and sham-tSCS). Both, participants and the assessor (AMG) were blinded, and the order for each recruited participant to receive either the sham or active tSCS intervention was randomized, with the consecutive order concealed in individual sealed envelopes. Randomization was performed using the web software www.randomizer.org. Intervention sessions were separated with a minimal 48 h washout period, which was longer than that used in similar studies (Aarskog et al., 2007; Buonocore and Camuzzini, 2007; Chen and Johnson, 2009; Dean et al., 2006).

In order to check the reliability of the neurophysiological outcomes, two measurements of quadriceps (Q) and tibialis anterior (TA) MEPs were recorded at baseline (PRE 1 and PRE 2) with a rest period of 2 min between them. Both Q and TA MEPs were recorded again during the intervention at 4 min from the onset of the tSCS intervention (DURING) and following the stimulus at 2 min (POST 1) and 4 min (POST 2). Postintervention measurements (POST 1 and POST 2) were recorded to determine the duration of the neuromodulatory effect following tSCS. Two measurements of isometric maximal voluntary contraction of the Q and TA muscles were recorded as secondary outcomes at PRE 1 and POST 2.

### 2.2. Procedures and Intervention

Fifteen healthy participants ≥ 18 years old, without injury to the central or peripheral nervous system, were recruited via non-probabilistic convenience sampling. The exclusion criteria for recruitment included musculoskeletal pathology of the lower limbs, metal or electronic implants, medications that influenced neural excitability (antiepileptic, antipsychotics, or antidepressants), allergy to the electrode material, epilepsy, and pregnancy.

Participants adopted a relaxed supine position without voluntary muscle contraction. Three self-adhesive surface electrodes (9 × 5 cm, ValuTrode, Axelgaard Manufacturing Co, Fallbrook, CA, USA) were used for tSCS. The anode was placed on the midline skin surface between the T11 and T12 spinous processes. Two interconnected cathodes were placed symmetrically on the abdomen, at both sides of the umbilicus. A rectangular foam (12 × 7 × 2 cm) was used to increase the pressure on the anode by strapping both the sponge and electrode around the torso, to improve skin contact. The electrical stimulator used for all interventions was an electrotherapeutical device (Enraf Nonius, Myomed 932, Rotterdam, The Netherlands).

#### 2.2.1. Transcutaneous Spinal Cord Stimulation (Active-tSCS)

A symmetrical biphasic 30 Hz current of 1 ms pulse-width was applied at the threshold stimulation intensity required to evoke the PRM reflex. To calculate the PRM threshold the same device, electrode location, and waveform of current were used. The stimulus intensity was gradually increased in steps of 1 mA every 1 s until a minimally visible contraction was visually detected in the Q muscle. If subjects could not tolerate the stimulus intensity required to elicit the threshold PRM reflex, the stimulus was reduced to the intensity which was comfortable. The therapist informed the participant that the intensity could be set below the sensory threshold, with the possibility that the participant may or may not feel the stimulation current tSCS was applied for a total of 10 min with the subjects relaxed in the supine position.

#### 2.2.2. Sham-tSCS

Sham stimulation consisted of the same stimulation parameters used for active tSCS except for the stimulus intensity, which was set at the sensory threshold, maintained for 30 s and then slowly decreased to zero, where the stimulus intensity was set for the remaining 10 min intervention. This method has been previously validated as an appropriate sham stimulus for controlled studies (Deyo et al., 1990; Petrie and Hazleman, 1985) [28,29]. The participants received the same instructions that given to the active tSCS session.

### 2.3. Blinding Assessment

Both, the intervention, and evaluation process of the protocol were carried out by two independent researchers (NCS and AMG, respectively).

The success of participant and assessor blinding were evaluated through a questionnaire [30] after each intervention (active-tSCS and sham-tSCS), by assessing whether they had received the sham or active tSCS with five questions: (1) “Strongly believe the applied intervention is new treatment”; (2) “Somewhat believe the applied intervention is new treatment”; (3) “Somewhat believe the applied intervention is a placebo”, (4) “Strongly believe the applied intervention is a placebo”, or (5) “Do not Know”.

### 2.4. Primary and Secondary Outcome Measures

#### 2.4.1. Motor Evoked Potential Recordings

Motor evoked potentials were elicited by transcranial magnetic stimulation (TMS, Magstim Rapid 2, Magstim Company Ltd., UK) using a double-cone coil. The optimal stimulation site (hot-spot, area where TMS elicited the largest MEP) was identified for both the Q and TA muscles individually with reference to the standard CZ point (approximately 1 cm lateral and 2 cm posterior to CZ). The coil was then fixed, and the rim of the coil was marked with a pen on the scalp, so that the stimulation site was maintained constant. MEP threshold was defined as the minimal TMS intensity required to evoke a motor response (>0.1 mV peak-to-peak amplitude) during slight tonic contraction of the target muscle (approximately 20% of the isometric MVC) [28,29]. Test MEPs during the protocol were recorded during 20% MVC as an average of in response to 10 single-pulse stimuli applied at 120% of the MEP threshold. A maximal voluntary contraction was performed individually for Q and TA to record 100% EMG activity for each muscle, so that the 20% EMG activity could be estimated and used as a visual feedback to instruct the subjects to maintain that level of muscle contraction. Once the subjects had learnt to contract either the Q or TA muscles at 20% maximal voluntary activation, TMS-evoked MEPs were performed. EMG was recorded using bipolar silver chloride electrodes (×1000 amplification) filtered with a built-in 20–450 Hz bandpass filter (Signal Conditioning Electrodes v2.3, Delsys Inc., USA). Electrodes were placed over the rectus femoris of the belly of Q muscle and the proximal third region TA following the SENIAM recommendations. Average MEP peak-to-peak amplitude and latency were analyzed as the primary outcome measure of tSCS.

#### 2.4.2. Isometric Maximal Voluntary Contraction Strength

Isometric MVC strength was measured using the hand-held dynamometer Micro Fet 2TM (Hoggan Scientific, LLC, Utah, USA,), a method with intra and inter-rater reliability has been reported [31]. Assessment of isometric MVC strength was performed with participants in either the sitting or supine position to evaluate Q and TA muscles,, respectively. Participants were instructed to hold the side of the table for stabilization and contract the test muscle as hard and as fast as they could against the dynamometer and to maintain the contraction until the assessor requested them to “stop”. Each test measured MVC strength during three to five seconds. Three test trials, with a 1 min interval resting period, were performed for each muscle group. Average peak force was used for statistical analysis.

### 2.5. Statistical Analysis

The sample size was calculated based on Q-MEP amplitude as the main variable. A mean difference of 0.5 mV (approx. 30%) between groups was expected, with a standard deviation of 0.065 was considered from a previous preliminary unpublished report, with a type I error (α) of 0.05 and a power of 80%. A sample size of 15 subjects was calculated.

Statistical analysis was performed using the commercial software package SPSS v22. Assessment of the relative reliability of baseline MEPs for PRE1-PRE2 was achieved with the intraclass correlation coefficient (ICC) that indicates the error in measurements as a proportion of the total variance in scores. An ICC of over 0.90 was defined as a high; from 0.80 to 0.90 as moderate; and below 0.80 as low reliability [32,33]. Moreover, the homogeneity between sessions for MEPs and PRM baseline threshold was analyzed with a t-student test. For statistical analysis of MEPs, the average of PRE1 and PRE 2 was considered as the unique baseline (PRE) measure. A two-way repeated-measures ANOVA, with “time” as one factor (PRE, DURING, POST1, POST2) and the “intervention” factor (tSCS and Sham) was performed to compare differences in MEP amplitude latency. Similarly, a two-way repeated-measures ANOVA was carried out for dynamometer data collected at PRE1 and POST2. A paired t-test comparison with a Bonferroni correction for multiple comparisons was used to highlight specific differences between time and interventions. To analyze the blinding outcome variable, James’ Blinding Index (BI) [34] and Bang’s BI [30] were obtained using Stata v15.0 (Stata Corp, TX, USA). James’ BI is used to infer the overall blinding success in RCTs. However, Bang’s BI is used to characterize and evaluate the blinding situation in each trial arm independently. James’ BI ranges from 0 to 1 (0 representing total lack of blinding, 1 representing complete blinding, and 0.5 representing completely random blinding). To interpret the results, this study considered a lack of blinding if the upper bound of the confidence interval (CI) was below 0.5. Bang’s BI can be directly interpreted as the proportion of the unblinding in each arm [30]. It ranges between −1 and 1, with 0 as a null value indicating the most desirable situation representing random complete blinding. Therefore, when one-sided CI did not cover the 0 value, the study was regarded as lacking blinding.

## 3. Results

**Figure 1:**
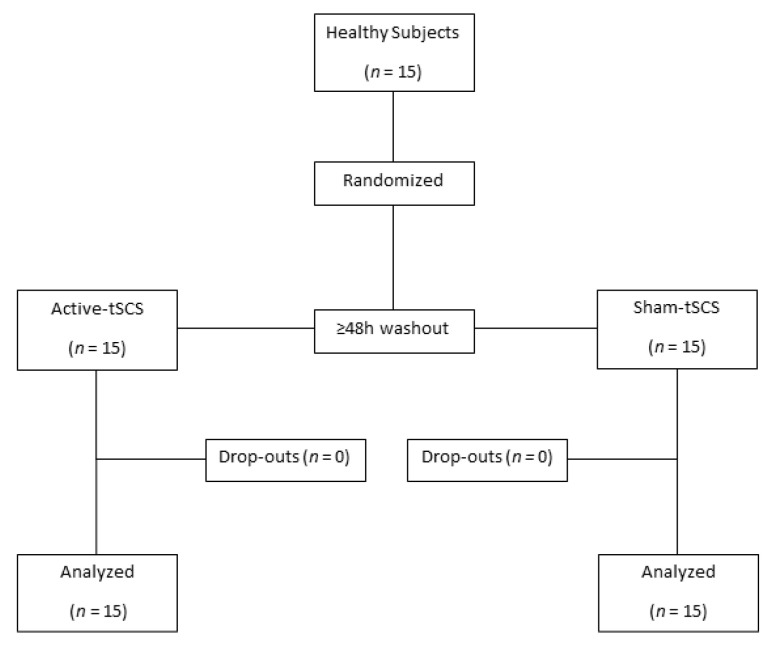
Consolidated Standards of Reporting Trials (CONSORT) flow diagram.

Fifteen participants were recruited and completed the study. Nine were female (60%) and 6 males, with a mean age of 25.2 years old (SD 3.8), weight of 60.9 kg (SD 12.62), height of 1.67 m (SD 0.11), and a mean body mass index of 21.6 (SD 2.47). None of the subjects withdrew from the study (Figure 1. CONSORT flow diagram). No sex significant differences were found when comparing the proportion of the subjects (*p* > 0.05) nor when comparing the effect of tSCS during the stimulation (*p* > 0.1) and after the stimulation (*p* > 0.2) for both Q-MEP and TA-MEP outcomes.

With regard to baseline neurophysiological parameters, mean MEP thresholds for Q (t = 0.00, *p* = 1.00) and TA (t = −0.187, *p* = 0.85), and PRM reflex threshold (t = −0.58, *p* = 0.56) were similar before application of sham or active tSCS (Table 1). The averaged tSCS intensity during the active-tSCS session was 27.6 mA (8.9), that was 27.5% less than the PRM reflex threshold (Table 1). Basal peak-to-peak amplitude MEP measured at PRE1 and PRE2 were highly reliable for both active and sham interventions. During the active tSCS session, a 0.91 ICC was registered for Q-MEP amplitude and a 0.97 ICC for TA-MEP amplitude. During the sham intervention, a 0.89 ICC was registered for Q-MEP amplitude and a 0.97 ICC for TA-MEP amplitude were calculated. Regarding Q-MEP latency, a 0.97 ICC was calculated during the active tSCS session and an ICC of 0.97 during sham intervention. The latency of TA-MEPs showed an ICC of 0.95 during active tSCS intervention and 0.92 during the sham session.

### 3.1. Effect of tSCS on Peak-To-Peak MEP Amplitude

**Figure 2:**
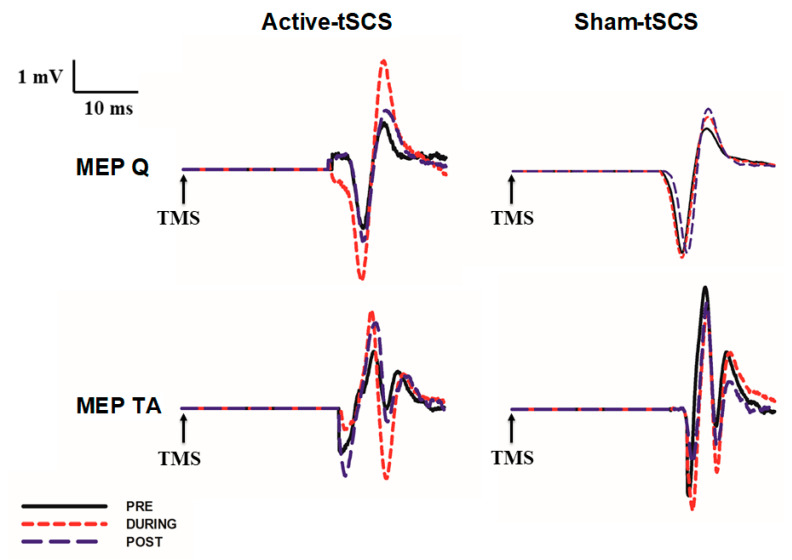
Representative motor-evoked potentials from participant #7 recorded from the Quadriceps and Tibialis Anterior muscles PRE, DURING and POST active or sham tSCS.

**Figure 3:**
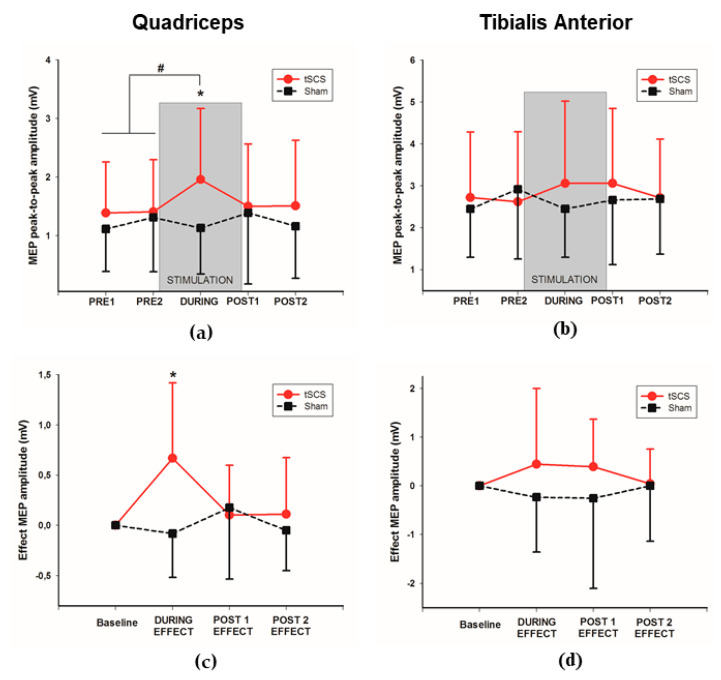
MEP peak-to-peak amplitude: (**a**) MEP of quadriceps muscle at each evaluation time; (**b**) MEP of tibialis anterior muscle at each evaluation time; (**c**) change obtained in MEP of quadriceps during and after tSCS respect to baseline; (**d**) change obtained in MEP of tibialis anterior during and after tSCS respect to baseline. * *p* ≤ 0.05 when compared to sham-tSCS; ^#^ *p* ≤ 0.05 when compared from baseline.

The effect of active or sham tSCS on MEPs recorded from subject (#7) (Figure 2) is illustrated on the Q and TA muscle (Figure 2), recorded at PRE 2, during the intervention (DURING), and after the intervention finished (POST 1). Mean peak-to-peak amplitude and the change in MEP amplitude DURING and POST tSCS are represented in Figure 3A–D, respectively. In the quantitative analysis, the two-way ANOVA showed no significant differences in Q-MEP amplitude for either the “intervention” factor (F = 1.178, *p* = 0.28) or the “time” factor (F = 1.176, *p* = 0.18). However, a significant “intervention-time” interaction was found for Q-MEP amplitude (F = 3.88, *p* = 0.02). A post-hoc pairwise comparison of Q-MEP amplitude revealed a significant increase during active-tSCS (1.96 mV; SD: 0.3) when compared to the averaged PRE amplitude (1.40 mV; SD: 0.2; *p* = 0.01). Furthermore, a higher amplitude of Q-MEPs during active-tSCS (1.96 mV; SD: 0.3) was observed when compared to sham-tSCS at the same time-point (1.13 mV; SD: 0.3; *p* = 0.03; Figure 3A). Regarding TA-MEP amplitude, although a greater amplitude during the active tSCS intervention (Figure 3B) was identified (3.06 mV; SD: 2.0) with respect to the PRE baseline amplitude (2.67 mV; SD: 0.4) and higher than the equivalent MEP amplitude during sham tSCS (2.45 mV, SD: 1.1), no statistical significant differences were observed for the factors analyzed (“intervention” F = 0.268, *p* = 0.609; nor “time” TA F = 0.339, *p* = 0.797; nor “intervention-time” interaction F = 1.137, *p* = 0.333).

When the specific effect of each intervention was calculated subtracting the baseline activity in each time-point (Figure 3C,D), the pairwise comparison between groups with the Bonferroni correction revealed a mean difference of 0.73 mV (SD: 0.24; *p* = 0.05) during the stimulation for the Q-MEP, without significant differences for the POST-1 (0.001 mV; SD: 0.19; *p* = 0.94) and POST-2 (0.25 mV; SD: 0.16; *p* = 0.13) time-points (Figure 3C). For the specific effect of the intervention over TA-MEP, a non-significant difference between groups of 0.91 mV (SD: 0.62; *p* = 0.15) was evidenced during the stimulation. Neither significant differences were found for the POST-1 (0.69 mV; SD: 0.62; *p* = 0.21) and POST-2 (0.33 mV; SD: 0.53; *p* = 0.53) time-points (Figure 3D).

### 3.2. Effect on MEP Latency

Table 2 reveals that the application of tSCS intervention had no effect on MEP latencies for the Q and TA muscles. The two-way ANOVA revealed no significant differences neither for the “time” factor (Q muscle: F = 1.952, *p* = 0.13; TA muscle: F = 0.89, *p* = 0.45), “intervention” factor (Q muscle: F = 0.549, *p* = 0.46; TA muscle: F = 2.621, *p* = 0.11), nor “intervention-time” interaction (Q muscle F = 0.937, *p* = 0.42; TA muscle: F = 0.254, *p* = 0.86).

### 3.3. Effect on Isometric Maximal Voluntary Contraction Strength

Table 3 shows the effect of tSCS on mean peak isometric MVC force recorded from the Q and TA muscles before and after the active and sham interventions. No significant differences were observed in MVC strength when pre-post values were compared for active-tSCS and sham-tSCS (Q: F = 0.583, *p* = 0.45; TA: F = 0.268, *p* = 0.60). In the same way, no significant differences were observed for the “time” factor (Q: F= 0.017; *p* = 0.89; TA: F = 0.339; *p* = 0.797) nor the “intervention-time” interaction (Q: F = 0.981, *p* = 0.33; TA: F = 0.808, *p* = 0.38).

### 3.4. Adverse Effects

No moderate or severe adverse events were reported in any of the participants. Thus, the stimulation was determined as a safe procedure. However, 66.6% (*n* = 10) of participants could not tolerate the optimal intensity previously determined (PRM threshold), considering the intervention as “very uncomfortable”. Two participants reported paraesthesia in lower limb during stimulation. Moreover, two patients reported DOMS (delayed onset muscle soreness) in quadriceps muscle evaluated 24 h after stimulation which could be generated by the voluntary isometric muscle contractions performed during the strength evaluation.

### 3.5. Blinding and the Assessment of Its Success

The guesses of the participants and assessor are shown in Table 4 in a 2 × 5 table format (Table 4). Furthermore, Table 5 shows James’ BI and Bang’s BI values obtained for the study subjects and assessor. According to the interpretation of data established by James et al. [34] and Bang et al. [30], when blinding was analyzed globally by James’s BI a lack of blinding was observed in participants and assessor. However, when each treatment arm (Active tSCS and Sham tSCS) was analyzed independently by Bang’s BI a successful blinding was observed for the participants and assessor in the sham group and the active group, respectively.

## 4. Discussion

The present study shows that a single session of 10 min of tSCS increases the peak-to-peak MEP amplitude of Quadriceps muscle, but without a change in muscle strength. Alpha-motoneurons located more distally below the level of tSCS, namely those innervating the TA muscle, did not show any change in MEP amplitude. This is the first study that describes an increase in corticospinal-evoked lower limb motor-evoked potentials after a single session of active-tSCS when compared to a sham tSCS. There is an urgent need to optimize the motor neuromodulatory effect of tSCS in subjects with SCI using neurophysiological measures that detect changes in both cortical and spinal excitability.

A number of studies in healthy individuals have observed that tSCS located over the T10-T11 vertebral level (L1-L2 spinal level) evokes multi-segmental responses from lower limb motoneurons [17,19,20,21]. However, both the excitation threshold and the motor response magnitude differ according to the specific positioning of the spinal stimulus [5,21,35]. Our study observed a significant increase of MEP amplitude in the Q muscle, which is innervated by the L1-L2 spinal level that corresponded to the level of tSCS. In contrast, the TA MEP amplitude, innervated by the L4-S1 spinal level, was not modulated by tSCS. Computational studies of the effect of tSCS suggest that afferent fibers with the lowest threshold are activated by spinal stimulation focused at the dorsal root entry zone, which is accessible via the intervertebral foramen [1,36,37,38]. In the case of TA, the afferents that optimally activate this muscle are located more distally to the site of the stimulating electrode. Another potential explanation for no change in TA MEP amplitude with tSCS is that the applied current intensity was insufficient to stimulate the afferents required to facilitate descending drive to the TA muscle, because the intensity was set with reference to the Q PRM reflex threshold. Previous studies have reported a higher PRM reflex threshold for the TA muscle [17,19,20,35]. In this study it is possible that the tSCS current intensity was not optimal to increase corticospinal drive to TA motoneurons.

Motor evoked potentials from the Q and TA muscles were recorded at 20% of MVC so as to reduce the variability due to changes in corticospinal excitability [39]. Knee extensor MEPs are reliably recorded in the active Q [40] in response to single pulse TMS, which permits measurement of MEP amplitude as a standard measure of corticospinal excitability [41]. This is the first study to identify a selective effect of tSCS on Q MEPs compared to distal lower limb muscles such as the TA. Recently, an effect of non-invasive spinal stimulation applied at 30 Hz pulses, with a 5 kHz carrier frequency, has been identified on MEPs recorded from the upper limb muscles following TMS in cases of cervical spinal cord injury [27]. Previously low frequency stimulation of afferents has been shown to increase corticospinal excitability in the upper limb [42]. In the current study tSCS was applied at 30 Hz at the spinal lumbar level, which led to an increase in CST excitability during spinal stimulation. The TA MEP also revealed a non-significant increase in amplitude both during and immediately after spinal stimulation.

Non-invasive spinal stimulation may activate several afferent systems which are known to modulate descending excitation of spinal systems involved in motor control. Activation of the skin of the anterior aspect of the thigh activates both excitatory and inhibitory spinal interneurons-including transcortical pathways directed to biceps femoris (BF) and Q muscles [43]. Activation of cutaneous afferents within the sural nerve have been shown to facilitate TMS evoked TA muscle activity [44]. Cutaneous afferents are also known to activate transcortical pathways as suggested in studies of people with dorsal column injury or corticospinal tract abnormalities [45], although activation of subcortical pathways may also be possible [27,46]. Cutaneous volleys evoke muscle responses at a transcortical latency followed by a longer-latency excitatory response [47,48] and ongoing voluntary contraction of lower limb muscles can also potentiate this longer-latency excitatory cutaneomuscular response [48].

A role for group I proprioceptive afferents in tSCS-mediated Q muscle facilitation is also possible, especially as spatial facilitation of corticospinal evoked responses is increased with TMS and simultaneous afferent activation following common peroneal nerve activation, mediated at an interneuronal site [49]. Furthermore, during weak voluntary contraction of the Q muscle, common peroneal nerve [50] or femoral nerve [51] group I mediated facilitation of the Q H-reflex was increased. These studies suggest that the main role of descending facilitation of feedback inhibitory interneurons to lumbar propriospinal neurons during weak Quadriceps contraction is to focus corticospinal control of a limited number of motoneurons required for muscle activation.

The increase of the Q MEP amplitude was first detected 4 min after the onset of the 10 min stimulation, and supports previous studies that have observed an immediate increase of voluntary EMG activity, voluntary movement, and gait function in subjects with spinal cord injury as soon as the current is switched on [8,52]. In our study, the increase in peak-to-peak Q and TA MEP amplitude was not maintained up to 2 and 4 min after tSCS. In contrast in a recent study a 20-min single session of tSCS increased the amplitude of subcortical motor evoked responses in arm muscles of people with a high level of SCI for up to 75 min [27]. Moreover, other studies have identified prolonged post intervention effects of tSCS based on improvement of spasticity [13] or the subjective perception of voluntary movement [8]. Other studies have identified improved upper arm function at 3–6 months after 20–30 repeated sessions of tSCS measured with handgrip strength, upper limb EMG activity and an improvement in general functionality (Upper Extremity Motor scores and Action Research Arm Test) [23,53]. These studies applied tSCS for greater than 20 min, which in addition to our study, supports the hypothesis that repeated tSCS interventions of longer than 20 min is required to elicit immediate postintervention effects that lead to an effective therapeutic option.

To date, the vast majority of studies have fixed the intensity of tSCS by using the subjective perception of sensations made by participants or more directly by detecting muscle contraction in response to the stimulus [6]. Recently, Serrano-Munoz et al. [54] demonstrated the practical limitations of adjusting the intensity of stimulation current based on subjective perception and highlighted the importance of defining objective parameters to adjust current intensity. In this study, PRM reflex threshold was used as an objective measure to adjust stimulation intensity. However, only 44% of the participants could tolerate this objectively defined intensity when tSCS was applied at this intensity at 30 Hz (see Table 1). The other participants received a more tolerable stimulus intensity based on their subjective tolerance threshold. Further protocols are necessary to explore a wide range of tSCS frequencies and intensities to improve the tolerance threshold of subjects in order to optimize the effect of neuromodulation on motor function.

The study was performed with the subject in a relaxed supine position, a posture which has been shown to be associated with the most effective tSCS neuromodulatory effect [55]. Indeed, currently the most optimal translation of the tSCS technique to clinical practice is to adopt supine-position stimulation strategies in combination with other activity-based neurorehabilitation tasks. However future studies should examine position-dependent tSCS efficacy using the MEP technique with the subject performing tasks such as sitting or standing, which are more compatible with actual rehabilitation techniques.

Although MEPs evoked by TMS have been demonstrated to be a reliable tool to quantify the excitability of the corticospinal pathway [40,56], it is questionable if MEPs are sensitive enough to reflect functionally relevant changes evoked by the tSCS. In the present study, both TA and Q MEPs were stable at baseline, and were reproducible when measured at the two pre-evaluation test periods. Stable Q MEPs recorded before tSCS together with the absence of changes during the sham intervention support the significant neuromodulatory effect of active tSCS on Q muscle activity. Furthermore, this finding supports the use of MEPs in future studies designed to optimize tSCS parameters including the specific location of the electrodes.

Sham-controlled interventions have been applied previously to control the effect of peripheral electrical stimulation [28,29] and electrical stimulation of the CNS [57,58]. To our knowledge this is the first study of tSCS where the success of the blinding procedure has been formally assessed using the specialized blinding questionnaire [30], which was completed by both the assessor and the participants. The sham protocol was evaluated as a good method to blind participants who were to receive the sham tSCS, but not when they received active intervention, because 73% of participants correctly guess the assignment. This issue may have arisen due to fact that the previous experience of participants with electrical stimulation was not controlled appropriately, possibly because of the perception of the high stimulus intensity. Previous studies of transcranial direct current stimulation (tDCS) have shown that the application of high intensity electrical stimulation hinders the blinding of participants [59,60]. In contrast, the current study has shown that the blinding procedure was evaluated as good when used to mask the assessor using the active tSCS intervention, but not with sham stimulus application, because the assignment was correctly guessed by the assessor in 73% of participants. Although James’s Blinding Index indicated that blinding was not generally effective as assessed by participants and the assessor, the upper 95% CI limit was 0.48 for participants and 0.40 for the assessor, close to the value of 0.5 which is defined as successful. Finally, it is necessary to highlight that crossover studies are inherently difficult to blind compared to more effective parallel design studies. Although further improvement in intervention blinding techniques are still required for the design of future randomized controlled trials, the current sham protocol based on applying only 30 s of active tSCS intensity appears to be a good option to blind both subjects and assessors to the intervention.

No serious side effects were observed after the single 10-min tSCS session in this study. In a recent systematic analysis of the effect of tSCS on motor function after spinal cord injury, this technique has been shown to be painless, well tolerated and safe [6]. However, in our study, eight participants reported discomfort around the electrodes when tSCS commenced. In most cases, this discomfort was related to a strong contraction of abdominal muscle generated by the electrical stimulation. For this reason, 10 participants could not tolerate the intensity of current previously set according to the PRM reflex threshold. Considering that the participants of this study received a single session of tSCS, it is possible that stimulus training sessions are necessary so that participants become accustomed to the tSCS and to improve tolerance. Thus, in a recent study which involved the self-administration of the tSCS applied over 6 week in subjects with spinal cord injury has been shown to be safe and well tolerated in a home-based setting [13]. Future studies should consider employing repeated sessions of tSCS where the intensity is increased incrementally to improve subject tolerance and therefore allowing higher current density and more effective neuromodulation.

## 5. Conclusions

A single 10 min session of active tSCS applied over the T11-T12 vertebral level increased the quadriceps muscle MEP but not for the TA muscle, when compared to sham stimulation. The neuromodulatory effect of the motor response was specific to the stimulation of the metameric level innervating the Q muscle. In this study we have shown that MEP amplitude is a reliable and sensitive technique to demonstrate changes in the excitability of the corticospinal tract after active tSCS. The used method for sham stimulation could be a feasible option for future tSCS interventions. More randomized sham-controlled clinical trials will be needed to optimize the efficacy of active tSCS to promote motor recovery in subjects with spinal cord injury.

## Figures and Tables

**Table 1:** Neurophysiological assessment at baseline time for each session.

	Active-tSCS Session				Sham-tSCS Session		
Subject	MEP-t Q (%)	MEP-t TA (%)	PRM-t (mA)	Intensity tSCS (mA)	MEP-t Q (%)	MEP-t TA (%)	PRM-t (mA)
01	55	50	45	45	55	55	41
02	31	31	40	25	32	32	40
03	42	35	29	29	35	35	29
04	45	40	33	33	55	44	32
05	50	50	33	33	55	55	33
06	42	42	35	26	40	40	30
07	32	32	50	37	30	30	40
08	32	32	40	21.5	45	35	43
09	47	42	35	14	37	35	29
10	35	35	35	27	33	38	35
11	42	37	41	39	38	33	45
12	35	35	40	17.5	34	34	40
13	38	35	38	22	38	35	38
14	35	35	36	13	35	35	26
15	54	40	44	32	53	42	46
Mean	41	38.1	38.3	27.6	41	38.5	36.5
(SD)	(7.9)	(5.9)	(5.2)	(8.9)	(9.1)	(7.6)	(6.3)

PRM = posterior root-muscle reflex; Q = quadriceps; SD = Standard deviation; −t = threshold; TA = tibialis anterior; tSCS = transcutaneous spinal cord stimulation.

**Table 2:** Latencies MEPs following active-tSCS or sham-stimulation. Mean and standard deviation (SD).

		PRE 1	PRE 2	DURING	POST 1	POST 2
MEP Q (ms)	Active-tSCS	21.74 (1.98)	21.71 (2.07)	21.77 (2.11)	21.81 (2.01)	21.72 (2.04)
	Sham-tSCS	22.49 (2.78)	22.53 (3.1)	22.70 (2.77)	22.35 (3.04)	22.48 (3.07)
MEP TA (ms)	Active-tSCS	28.83 (1.81)	29.03 (1.7)	28.71 (1.75)	28.77 (1.79)	28.72 (1.67)
	Sham-tSCS	29.54 (2.66)	29.97 (1.97)	29.92 (2.27)	29.72 (2.00)	29.80 (1.86)

**Table 3:** Isometric maximal voluntary contraction strength following tSCS or Sham. Mean (SD).

	Active-tSCS			Sham-tSCS		
	PRE	POST	% of Change	PRE	POST	% of Change
Strength Q (Kgs)	76.18 (16.62)	77.91 (18.38)	3.10 (13.23) *p* = 0.428	82.84 (17.84)	81.30 (21.50)	−2.32 (11.47) *p* = 0.562
Strength TA (Kgs)	73.46 (11.96)	73.00 (14.69)	−1.09 (6.43) *p* = 0.699	82.84 (17.84)	81.30 (21.50)	1.55 (6.11) *p* = 0.392

Kgs = kilograms; POST = postintervention; PRE = preintervention; Q = quadriceps; SD = standard deviation, TA = tibialis anterior.

**Table 4:** Assessment of the blinding. Absolutes values and percentages of answers selected by participants and assessor.

**Assignment**	**Participants’ Guess, *n* (%)**					
	**Strongly** **Active tSCS**	**Somewhat** **Active tSCS**	**Strongly** **Sham tSCS**	**Somewhat** **Sham tSCS**	**Do not Know**	**Total**
Active tSCS	9 (30.0)	4 (13.3)	0 (0)	0 (0)	2 (6.7)	15 (50.0)
Sham tSCS	4 (13.3)	4 (13.3)	2 (6.7)	4 (13.3)	1 (3.3)	15 (50.0)
Total	13 (43.3)	8 (26.7)	2 (6.7)	4 (13.3)	3 (10.0)	30 (100.0)
**Assignment**	**Assessor’s Guess, *n* (%)**					
	**Strongly** **Active tSCS**	**Somewhat** **Active tSCS**	**Strongly** **Sham tSCS**	**Somewhat** **Sham tSCS**	**Do not Know**	**Total**
Active tSCS	3 (10.0)	6 (20.0)	1 (3.3)	3 (10.0)	2 (6.7)	15 (50.0)
Sham tSCS	0 (0.0)	0 (0.0)	4 (13.3)	9 (30.0)	2 (6.7)	15 (50.0)
Total	3 (10.0)	6 (20.0)	5 (16.7)	12 (40.0)	4 (13.3)	30 (100.0)

**Table 5:** Statistical analysis of blinding assessment.

**Participants Results**				
**Methods**	**Index**	***p*** **-Value**	**95% Confidence Interval**	**Conclusion**
James	0.36	0.031	0.24 to 0.48	Unblinded
Bang-Active/2 × 5	0.73	<0.001	0.58 to 0.89	Unblinded
Bang-Placebo/2 × 5	−0.07	0.63	−0.40 to 0.27	Blinded
**Assessor Results**				
**Methods**	**Index**	***p*** **-Value**	**95% Confidence Interval**	**Conclusion**
James	0.27	0.001	0.14 to 0.40	Unblinded
Bang-Active/2 × 5	0.17	0.18	−0.13 to 0.46	Blinded
Bang-Placebo/2 × 5	0.73	<0.001	0.58 to 0.89	Unblinded
